# Relationships between Agile Work Practices and Occupational Well-Being: The Role of Job Demands and Resources

**DOI:** 10.3390/ijerph19031258

**Published:** 2022-01-23

**Authors:** Sarah Rietze, Hannes Zacher

**Affiliations:** Department of Work & Organizational Psychology, Wilhelm Wundt Institute of Psychology, Leipzig University, 04109 Leipzig, Germany; hannes.zacher@uni-leipzig.de

**Keywords:** agile work practices, work characteristics, job demands, job resources, occupational well-being

## Abstract

Agile work practices have been adopted by most software development organizations and by many large organizations from other industries. The introduction of agile work practices is assumed to positively affect work characteristics and, in turn, well-being of employees. So far, there is only very little and methodologically limited empirical research on this topic. Based on job demands–resources theory, we developed and tested a model on the direct and indirect relationships between agile work practices, job demands and resources, and occupational well-being. Data were provided by 260 employees working in agile development teams who participated in two surveys that were approximately six weeks apart. Results of structural equation modeling provided support for the hypothesized model, suggesting that agile work practices have a negative indirect effect on emotional fatigue through lower job demands. At the same time, agile work practices also had a positive indirect effect on emotional engagement through higher job resources. Our research contributes to the literature by integrating agile work practices with job demands–resources theory, bridging an important gap between research and practice. Overall, the findings suggest that the implementation of agile work practices may have a positive impact on occupational well-being by improving employees’ perceptions of key work characteristics.

## 1. Introduction

Agile work seems to be “the new Holy Grail” for organizations [1]. Organizations recognize the need to implement agility in their structures and processes to keep pace with today’s complex, challenging, and competitive business environment [2]. Some key characteristics of agile work include self-organized teams, quick and proactive decision-making, transparency, collaboration, regular reflection within the team, customer centricity, and an iterative work approach [3]. Agile work practices were first established in software development to better meet customers’ requirements and to increase productivity and quality. To this end, an incremental development instead of a heavyweight plan-driven approach is adopted, and self-management of teams is enhanced [4]. Moreover, increased proactivity in agile teams seems to be an important influencing factor for the positive impact of agile taskwork on team performance [5].

Although agile work practices have received much research attention in the last twenty years, the role of agile work practices in occupational well-being is currently not well understood. There are two main reasons for this lacuna. First, previous research is mainly anecdotal and, thus, does not allow for valid conclusions [6]. This research is also criticized for lacking a solid theoretical ground [7,8] and for being largely based on small samples from only one industry or company [9]. Second, the assumption of a positive impact of agile work practices on well-being has so far mostly been argued from a practical perspective, published only in the software development literature [10]. In contrast, psychological research has not yet addressed the issue of agile work practices and their effects, maybe because of unfamiliarity with the software technology literature, or because psychologists might believe that agility is nothing new or just a fast-fading trend. However, agile work practices are becoming more widespread, and they involve fundamentally different approaches to team collaboration than traditional project work.

**Figure 1:**
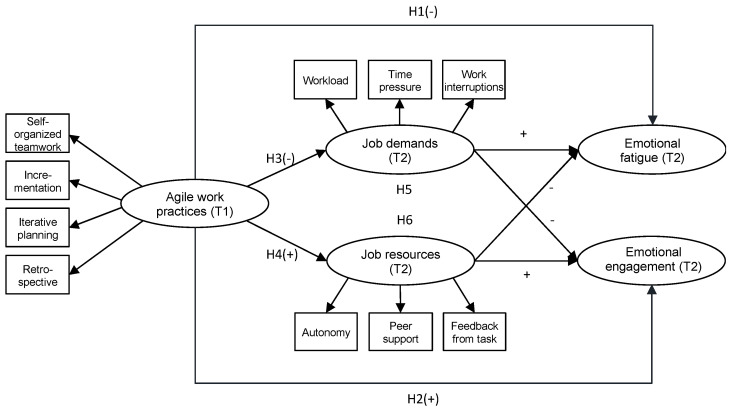
The hypothesized job demands–resources model for agile work practices.

According to self-determination theory [11], meeting the innate psychological needs for autonomy, belonging, and competence promotes intrinsic motivation, self-regulation, and well-being in individuals. Agile work provides an environment that supports the fulfillment of those needs by fostering important job resources [9,12] and psychological empowerment [13]. In addition, agile work practices have the potential to decrease stressful demands by promoting a sustainable pace [14], by fostering self-organization [15], and by offering a safe space to team members [16]. Agile work practices may relate to employees’ perceptions of work design and work characteristics [8,12,17], which in turn are important predictors of occupational well-being [18]. In this paper, we develop and test a model to better understand the link between agile work practices, work characteristics, and occupational well-being. Moreover, we combine agile work practices with a well-established psychological theory of work characteristics and well-being, the job demands–resources (JD–R) theory [19]. The JD–R approach accounts for two main mechanisms: first, the energy-depleting process triggered by job demands, which have a negative impact on well-being; and second, the motivation process started by job resources, which have a positive impact on well-being. We investigate the extent to which agile work practices relate to occupational well-being via the two processes of well-being described above. Our conceptual model is shown in Figure 1. Overall, we assume that agile work practices positively relate to occupational well-being. More specifically, we hypothesize that

- Agile work practices are directly related to lower levels of emotional fatigue and higher levels of emotional engagement, which represent two important indicators of occupational well-being [20,21];
- Agile work practices are directly related to lower levels of job demands and higher levels of job resources; and
- Agile work practices indirectly relate to occupational well-being via job demands (the energy-depleting process) and job resources (the motivation process).

This paper aims to contribute to the existing literature in several ways. First, although research on the consequences of agile work practices and research on effective work design should be closely related, they have so far largely been discussed separately (e.g., agile work practices in the software development literature and work design in the psychology literature). We integrate these two areas and explore the links between agile work practices, work design, and well-being based on well-established psychological theory and an empirical study. Second, we expand the JD–R literature by integrating concrete work practices (i.e., agile work practices) and investigating their role in both the motivational and energy-depleting pathways to occupational well-being. So far, research on JD–R theory focuses on individuals’ perceptions of work characteristics as predictors of well-being but has neglected the role of specific work practices implemented at team or organizational levels [22]. With the investigation of agile work practices in this context, we apply psychological theory to understand current practical issues and thus contribute to closing the gap between research and practice. Third, this paper also provides an important practical contribution for the many companies undergoing agile transformations. We provide insights into which resources need to be strengthened when introducing agile work practices and which potential demand traps organizations need to watch out for in order not to jeopardize the well-being of their employees.

## 2. Theoretical Background

### 2.1. Agile Work Practices

Agile work practices can be defined as project management and team practices that are based on a worldwide-agreed value system as described within the agile manifesto [4]. This manifesto offers an alternative to traditional ways of collaboration. Elements of the agile value system are designed to promote transparency, inspection, and adaptation [23]. Agile work practices are developed to directly address the problems of rapid change and to enable the team to respond effectively to change by streamlining information and decision-making processes [7,24]. Customers are not defined as mere recipients of the final product, but they are actively involved in the development process [4]. The business benefits of implementing agile work practices are diverse, such as team effectiveness, product quality, project performance, customer satisfaction, and project predictability [10,25,26]. Different agile methods and practices exist, such as Scrum, Kanban, or Extreme Programming, which all share common characteristics [10,27]. 

The focus of agile work practices is on the people who work together in a team [28]. The traditional command-and-control management style is replaced by a collaborative, self-organized management of the team itself [29]. This brings decision-making authority directly to the level of operational problems and uncertainties, resulting in increased speed and accuracy of problem resolution [30]. The role of management is to provide an environment of support and trust and to act as a servant leader. 

For this study, we focused on four key agile work practices that make up the core elements of agile working, namely self-organized teamwork [31], iterative planning, incrementation, and retrospective [5,12].

#### 2.1.1. Self-Organized Teamwork

Team self-organization means that agile teams autonomously coordinate their own work and regulate their own boundary conditions, and that team members share leadership and decision authority [31]. This includes the commitment and willingness of team members to manage, structure, and organize themselves [3]. Agile project teams can freely choose tools and technologies as needed, and they can make personnel decisions as well as decisions about how to handle changes of user requirements on their own [32]. 

#### 2.1.2. Iterative Planning

Agile teams work in short, recurrent, one- to five-week-long work iterations. At the beginning of each iteration, team members and business owners agree on what will be delivered during the upcoming iteration [14]. The team estimates the amount of work each task will require, and, based on this, it plans and decides on how much work can be completed [12]. Agile teams prefer to reduce the scope of tasks to keep their timetables rather than delay deadlines [14]. Subgoals are defined regularly and evaluated at short intervals to be able to integrate customer feedback constantly. 

#### 2.1.3. Incrementation

Agile teams follow a process of incremental planning and delivery, aiming to deliver a potentially working product increment after each iteration to receive immediate feedback [12]. Projects are developed in a stepwise manner to achieve continuous improvements [3]. Furthermore, the work performed during each cycle is strictly defined so that major tasks need to be split into smaller components to fit with the length of the cycle [32]. Following an incremental approach allows agile teams to experiment with different ideas and to start developing prototypes that will be refined throughout each iteration [5]. 

#### 2.1.4. Retrospective 

Reflection is another key dimension of agility. Agile teams regularly question their behavior, reflect on their collaboration, and look for improvements in their work [3]. This reflection is usually done in regularly scheduled retrospective sessions in which the team looks back at the last iteration. Thus, retrospectives can be seen a structured way to foster team reflexivity [5]. Important aspects the team should focus on during the retrospective include the identification and discussion of obstacles, the discussion of feelings, team dynamics and conflicts, the analysis of previous action points, and the identification of future action points [33].

### 2.2. Occupational Well-Being and Job Demands–Resources Theory

Subjective well-being refers to how a person values their life [34]. A person has high subjective well-being when, first, they are satisfied with their life (cognitive level) and, second, they frequently experience positive emotions such as joy or happiness and rarely experience negative emotions such as sadness or anger (affective level). Subjective well-being can vary across different life domains (e.g., family, work); however well-being in different life domains is highly interconnected [35]. Regarding the work domain, reciprocal relationships were found between life satisfaction and job satisfaction as well as between happiness in life and happiness at work [36]. Applying Diener’s definition [34] to the workplace, occupational well-being is composed of a cognitive evaluation of the job, such as job satisfaction, and an affective evaluation, meaning the positive or negative emotions employees experience at work, such as work engagement or burnout. 

JD–R theory [19,37] is one of the leading models that specify the work characteristics that are related to the affective well-being of individual employees. It provides a comprehensive theoretical framework that links aspects of work design with indicators of occupational well-being, such as exhaustion and engagement. According to the model, work characteristics, as antecedents of well-being, can be grouped into two major categories, job demands, and job resources. Job demands are work characteristics that require a high degree of physical, emotional, or mental effort. Job resources are health-protecting factors that stimulate personal growth and facilitate coping with high job demands [19]. JD–R theory proposes two main pathways to explain well-being. First, the confrontation with high or poorly designed job demands (e.g., time pressure, workload, or role conflicts) can lead to strain and exhaustion through a psychological stress process. A permanently high effort without regeneration possibilities leads to a full consumption of resources that can hardly be rebuilt. Second, the presence of high job resources plays an important intrinsic and extrinsic motivational role and leads to high work engagement [37]. Schaufeli and Taris [38] provide a comprehensive overview listing 61 job demands, job resources, and personal resources as antecedents of burnout and engagement as well as consequences of burnout and engagement, which have been extensively studied in JD–R research. Their meta-analysis shows that the scope of the JD–R study and the relationships assumed can be replicated for different countries, cultures, and occupational groups, including studies that consider software development environments. In summary, JD–R theory provides a solid foundation for exploring our questions about well-being in agile development teams.

## 3. A Job Demands–Resources Approach for Agile Work Teams

### 3.1. Total Effects of Agile Work Practices on Occupational Well-Being

Agile working may influence not only employees’ performance, but also their satisfaction and well-being at work, because people are put at the center of agile teamwork [28]. For example, agile team practices such as retrospective meetings or daily stand-ups lead to continuous exchange, shared learning, and high bonding within the team, increasing employee perceptions of psychological safety, transparency, and fairness of communication [39]. In addition, individuals in agile teams report high levels of psychological empowerment resulting from both regular team interaction and support, and greater team responsibility [32]. In agile teams, structures with fewer hierarchies, higher levels of autonomy, and increased team motivation are created, which in turn can lead to a higher level of job satisfaction [12,40,41], affective organizational commitment [9], and work engagement [42,43]. Furthermore, by promoting agile work practices within the organization, attractive and people-oriented values such as respect, courage, and commitment come into sharper focus and change the way team members feel [23]. Finally, agile teams are supported to focus on their core work and to experience the value they contribute to the big picture, which is positively associated with well-being [16].

At the same time, agile work practices may also prevent negative feelings and negative indicators of well-being at work, such as stress [15], overload [14], or exhaustion [17]. For example, an iterative approach not only enables a rapid response to changes but can also ensure a sustainable pace of work through balancing the workload during a work period [14]. In addition, the iterative process provides the opportunity to regularly experience success and minimize the risk of major failures [16]. Furthermore, a high level of team autonomy and consultation in decision-making can reduce stress at work [44] and is negatively related to exhaustion [45]. For example, by planning the tasks together before the next iteration, the team can have a good, shared estimate of how much work is realistically achievable, which can prevent overload and create regeneration potential between iterations [16]. To conclude, we assume an overall positive effect of agile work practices on well-being.

**Hypothesis** **1** **(H1).** *Agile work practices are negatively related to emotional fatigue.*

**Hypothesis** **2** **(H2).** *Agile work practices are positively related to emotional engagement.*

### 3.2. Direct Effects of Agile Work Practices on Job Demands

Job demands usually become stressors when meeting them requires too high effort or when individuals feel overloaded [19]. Agile teamwork is organized in a way that it has potential to prevent individuals from experiencing too high job demands. Agile teams have high levels of control over the planning and execution of their work, which, following the JD–R theory, can buffer the relationship between job demands and strain. More specifically, agile teams work in short iterations, for which they plan their workload in a self-determined manner and can thus influence that the scope of tasks and deadlines so as to keep them realistic. A clear prioritization for each defined iteration helps team members to work in a more concentrated manner and thus experience less interruptions. Workload, time pressure, and work interruptions are job demands that are linked to well-being at work, especially to its negative aspects, such as fatigue or burnout [37]. Thus, they are assumed to also play an important role in explaining well-being in the context of agile teams.

#### 3.2.1. Workload

Workload can become a burden if the ratio between the capabilities or resources available and the amount of work required for a task are disproportionate [46]. Agile teams plan and schedule their work themselves during planning meetings. Thus, they are in control of their own workload and pace of work [30]. Furthermore, perceived group autonomy is negatively linked to individual workload [47]. It was shown that a better balance of workload after the introduction of an agile method led to lower stress levels in the teams [48]. Furthermore, it can be assumed that agile work practices help to deal with high challenges, pressure, and stress by reducing complexity and ensuring continuity in the work progress. Finally, retrospective meetings also provide a platform to address workload issues and find appropriate solutions within the team. 

#### 3.2.2. Time Pressure 

The main stressors in traditional IT project teams include time pressure due to ongoing project deadlines [49,50] and unplanned time requirements [51]. Time pressure has a hindrance and health-impairing effect on employees if they feel that the pressure situation cannot be influenced [52,53]. Agile teams are responsible for their own work planning, so they have full control over how much work they put into a work iteration. The iterative approach allows requirements to be collected and planned for the team in a bundled way. This creates free working periods for the development team during an iteration. It has been shown that deadlines in highly agile teams are not perceived as a negative type of time pressure that causes stress, but that agile working promotes a healthy, sustainable pace within teams [14]. Furthermore, it is typical for agile work that each meeting is conducted according to a specific “timebox”, meaning that a planned activity takes place in a fixed and maximum unit of time. For example, a retrospective meeting should not exceed 90 minutes for a two-week iteration [23]. Timeboxing ensures that meetings remain focused and are not unexpectedly lengthened by discussions, which prevents developers from working productively. 

#### 3.2.3. Work Interruptions 

Work interruptions, for example by colleagues or phone calls, are also one of the key psychological strains found in traditional IT project environments [51]. On the contrary, working in a focused way by strictly following an iterative approach is one of the core agile values [23]. At the beginning of an iteration, teams jointly plan their priorities and agree on what will be delivered during the iteration. Once these priorities are transparent and committed, individuals can work through the tasks in a focused manner and are not disturbed by constant changes in scope [14]. The iterations can thus be understood as a protected space in which the individuals can concentrate on their tasks with few interruptions.

**Hypothesis** **3** **(H3).** *Agile work practices are negatively related to job demands (i.e., workload, time pressure, and work interruptions).*

### 3.3. Direct Effects of Agile Work Practices on Job Resources

The introduction of agile work practices in the workplace changes the way people coordinate, communicate, and work together in a team [54]. For example, iterative planning and retrospective meetings provide a platform for regular direct exchange and alignment. The meetings enable the team to keep each other informed, to solve problems together, to exchange feedback, and to discuss possible improvements, which helps the team to work more efficiently [55]. Furthermore, agile work practices support the feeling of belonging and the building of supportive relationships [56]. Shared leadership and self-management enable and motivate individuals through perceived autonomy and decision-making competence [54]. Thus, it can be assumed that agile work practices are positively related to important resources in the workplace. Self-determination theory (SDT) [11] provides a theoretical framework to explain why resources and consequently well-being should be particularly high in agile contexts. SDT assumes that human beings can be proactive and self-motivated or rather passive and disengaged, depending on the social environment in which they develop. Findings have revealed three innate psychological needs that enhance self-motivation and healthy psychological development. The three needs are (1) the need for autonomy, (2) the need for social belonging and support, and (3) the need for experiencing competence, which mainly comes from receiving feedback. Agile collaboration builds on these three innate needs, particularly by fostering key job resources such as autonomy, peer support, and feedback from the task.

#### 3.3.1. Autonomy 

Job autonomy is the “degree to which the job provides substantial freedom, independence and discretion to the individual” [57] (p. 258). It constitutes one of the most important job resources influencing employees’ well-being and job satisfaction [46,58]. Autonomy is also one of the key resources associated with self-organized, agile teamwork [9,12,17]. Autonomy can be divided into three different forms, which are decision-making, work method, and work scheduling autonomy [59]. Individuals in agile teams have a high degree of all three forms of autonomy, namely, to structure their own work process, to choose their own working methods, and to make their own decisions [30]. For example, the team plans and decides together how much work they can take into the next iteration and thus, takes responsibility for achieving the iterations’ goals [60]. Furthermore, agile meeting rituals, such as iterative planning and retrospective, also enhance participation, and team members are empowered to shape problem-solving processes [61]. During the iteration, the team self-organizes when and how they do their tasks. One goal of regular reflection after each iteration is to independently scrutinize processes and task performance and to develop and proactively address suggestions for improvement. Hence, individuals in agile teams should feel in control of their own behaviors. 

#### 3.3.2. Feedback from Task 

Feedback from tasks refers to the “degree to which carrying out the work activities required by the job results in the individual obtaining direct and clear information about the effectiveness of his or her performance” [57] (p. 258). It can be considered as another important resource that contributes to the success of agile work [17]. Agile work practices, for example, working in iterations and regular team reflection, are designed to provide regular opportunities to share knowledge, address difficulties, and exchange feedback on several perspectives [12,62]. For example, in retrospective sessions, each team member gives feedback on what worked well, what did not work well, and how things could be changed in the next iteration [23]. Some teams increase the visibility of the achieved performance by working with quantitative feedback using specific performance metrics, such as burndown charts. Furthermore, at the end of each work iteration, the agile team demonstrates the results in so-called review meetings to receive feedback from various internal and external stakeholders on the achieved product increment. Regarding the many different feedback opportunities, it can be assumed that members from agile teams perceive high levels of feedback from their work. 

#### 3.3.3. Peer Support 

“A supportive climate is characterized by coworkers who provide emotional comfort and serve as sympathetic listeners, a feeling that similar values are shared […], and a sense of respect for each other” [63] (p. 150). Agile, self-organized teamwork requires and promotes strong cooperation, communication, and mutual support within the team as team members share leadership and decision authority [60]. Social support can be foremost seen as a strategy for survival and success in an autonomous team setting. Consultation in decisions and teamwork are positively related to social support [44]. Agile work practices seem to improve both informal and formal communication [64]. Through frequent interaction, team members become more familiar with each other, which leads to an increased level of trust and helps workers to feel more comfortable asking for support [60]. In addition, agile work practices promote not only team-wide exchange and frequent interaction, but also the maintenance of team awareness of individual activities, which reinforces the feeling of acceptance and belonging to the team [56]. Failure to complete a task is not seen as an individual’s fault, but as collective responsibility, since everyone was involved in solving the problem in some way. Everyone can feel confident that their work is validated by the group, which increases the perception of help, trust, and goodwill. Finally, retrospective meetings as a forum to reflect on and improve team processes also foster peer support.

**Hypothesis** **4** **(H4).** *Agile work practices are positively related to job resources (i.e., job autonomy, feedback from task, peer support).*

### 3.4. Indirect Effects

Above we have argued that agile work practices should directly affect work characteristics, specifically job demands and job resources. Extensive research on JD–R theory shows that job demands are related to negative facets of well-being, job resources to positive facets of well-being, and that “cross-over effects” of demands and resources on these outcomes are also supported [38]. Additionally, recent studies focused on the role of work characteristics for well-being in the context of agile teamwork. For example, it was shown that autonomy and feedback of job resources are positively related to agile project management practices and job satisfaction [12]. Furthermore, agile work practices were found to be associated with team members’ perceptions of work autonomy and supervisor support as well as affective organizational commitment [9]. However, indirect effects of agile work practices on employee outcomes via work characteristics have not yet been shown, nor have different well-being outcomes and the two pathways been examined simultaneously. Following the dual-path approach of JD–R theory, we assume that the associations between agile work practices and well-being can largely be explained by work characteristics. More specifically, we suggest that, on the one hand, agile work practices have a stress-mitigating potential for agile team members by indirectly influencing emotional fatigue through lower job demands, such as workload, time pressure, and work interruptions. On the other hand, agile work practices have a motivational potential by indirectly influencing emotional engagement through higher job resources, such as autonomy, peer support, and feedback.

**Hypothesis** **5** **(H5).** *Agile work practices have a negative indirect effect on emotional fatigue via lower job demands.*

**Hypothesis** **6** **(H6).** *Agile work practices have a positive indirect effect on emotional engagement via higher job resources.*

## 4. Material and Methods

### 4.1. Procedure and Participants 

We tested the proposed model by asking individuals from the relevant population of professionals working in agile development teams to participate in an online survey. Study participants were contacted primarily through the professional networking platform LinkedIn. To take part in the study, participants were required to work for at least one year in an agile development team. Participants who had taken the first survey were contacted again after approximately six weeks and asked to complete a second survey. Agile work practices were measured at Time (T) 1, whereas work characteristics and occupational well-being outcomes were measured at T2 to minimize common method bias [65]. When predictor and outcome variables come from only one data source and are collected at a single point in time, the risk of obtaining an artificially inflated correlation between these variables is very high due to the same methodology underlying both measurements. The risk of such bias can be reduced by temporally separating measurements.

Baseline (T1) measures of work characteristics and well-being outcomes were also collected to examine effects of agile work practices on T1–T2 changes in these variables in supplemental analyses. Out of a total of 3534 people who clicked on the link in the invitation message, 700 people completed the first survey and 413 persons completed the second survey. A sample of 318 people participated in both surveys and provided usable and complete data. Furthermore, because we investigated Scrum work practices [23] in our study, e.g., iterative planning or retrospective, we included only those participants who work at least to some part with Scrum methodology compared to other agile methods. The final sample entails 260 participants, including 125 Scrum master or agile coaches (48.1%), 74 developers (28.5%), 46 product owner or product managers (17.7%), and 15 participants in other functions in agile teams (5.7%). Most of the participants were men (70.4%) and working in the German-speaking market (67.4% from Germany, Switzerland, or Austria). Most of the participants were younger than 40 years old (64.6%) and worked in large enterprises with more than 300 employees (64.1%). Most participants stated that they use Scrum (86.9%), and only a few indicated that they use a hybrid form of Scrum (Scrumban: 11.2%, Scrum/XP: 1.9%). However, only 13.1% stated that they use the method as it is theoretically defined. The rest indicated that they adapted the method slightly or strongly for their own needs. Most of the participants had more than two years of experience working with agile working methods (82.3%).

### 4.2. Measures 

Self-reported measures were collected via online surveys. Participants could choose between an English or a German form of the questionnaire. If validated translated scales were not available, items were translated from English or German using the forward–backward translation method. We examined the differences in variables between the English and German questionnaires. There were significant mean differences between the two questionnaires for the main variables self-organized teamwork (t(258) = 3.2, *p* < 0.01), incrementation (t(258) = 2.6, *p* = 0.01), and peer support (t(251) = 3.1, *p* < 0.01). For this reason, we conducted a supplemental analysis of an extended model in which we included language as a control variable (see Table S4 from https://osf.io/xpzbq (accessed on 22 December 2021)). All items were scored on 5-point Likert scales ranging from “strongly disagree” to “strongly agree.” All reliability estimates were acceptable and are shown in Table 1. The complete questionnaire can be seen in Table S1 from https://osf.io/xpzbq (accessed on 22 December 2021).

#### 4.2.1. Agile Work Practices (T1)

We used scales developed by Tripp and colleagues [12] to measure iterative planning and retrospective (three items each). A sample item for the 3-item subscale iterative planning is “The team estimates the amount of work each feature will require to be completed”. The subscale retrospective included items such as “On a regular basis, the team reflects on previous work and looks for ways to improve team performance”. Incrementation was measured with a 3-item subscale developed by Tuomivaara and colleagues [14]. A sample item is “At the end of each working period, we deliver a potentially shippable product to the customer”. Finally, the 3-item subscale self-organized teamwork was self-developed following the definitions and items of Stettina and Heijstek [31] and Moe, Dingsøyr, and Røyrvik [29]. An example item is “Each team member is involved in decision-making processes”. 

#### 4.2.2. Occupational Well-Being (T1 and T2) 

Occupational well-being was measured with three items each from the scales emotional work fatigue [21] and emotional work engagement [20]. Example items are: “At the end of the workday I often feel emotionally worn out” (i.e., emotional fatigue) and “I feel energetic at my job” (i.e., emotional engagement). 

*Job Demands (T1 and T2).* Job demands were measured using the scale workload from the Workload Inventory [66], as well as the two scales, work interruptions and time pressure, from the German instrument for Stress-Oriented Task Analysis [67]. All scales consisted of three items selected based on the highest factor loadings from the original scales [68]. Sample items include: “In my work there is often a great deal to be done” (i.e., workload), “It often happens that I have to interrupt current work because something important comes up in between” (i.e., work interruptions), and “My work often requires me to work very fast” (i.e., time pressure). 

#### 4.2.3. Job Resources (T1 and T2) 

The job resources autonomy and feedback from the job were measured using 3-item scales from the English and German Work Design Questionnaire [59,69]. A sample item for feedback from job is “The job itself provides feedback on my performance”. We measured autonomy using the means of the three subscales of job autonomy as indicators of a latent job autonomy construct, including work scheduling autonomy (e.g., “The job allows me to make my own decisions about how to schedule my work”), decision-making autonomy (e.g., “The job allows me to make a lot of decisions on my own”), and work methods autonomy (e.g., “The job allows me to make decisions about what methods I use to complete my work”). We measured peer support following the scale developed by Haynes and colleagues [70] selecting three items based on factor loadings, including, for example, “I can count on my colleagues to back me up at work”. 

#### 4.2.4. Control Variables 

For supplemental analyses, we measured high-performance work systems (HPWS) at T1 with nine items from the 10-item scale developed by Etchegaray and Thomas [71]. A sample item is “Employees in my organization are provided opportunities to learn new skills.” HPWS is “a group of separate but interconnected human resource practices designed to enhance employees’ skills and effort” [72] (p. 1069), such as selective hiring, extensive training, high-quality work, or information sharing [73]. HPWS were found to positively influence a variety of occupational outcomes related to well-being, for example, job satisfaction, psychological empowerment, or work engagement [74,75]. We assume that agile work practices are associated with work characteristics and well-being beyond HPWS, as it involves a new form of collaboration with new qualities, for example, self-organization or incrementation, which are not yet considered in traditional HPWS contexts. The wording of the HPWS items by Etchegaray and Thomas [71] was adapted to change the context from a hospital to organizations overall. For the same reason of context, one item was excluded from our scale (“Teamwork is important for providing quality service to patients”). 

### 4.3. Data Analysis

Statistical analyses were performed using RStudio [76]. We tested our models using structural equation modeling (SEM) and the “lavaan” package of the R statistical computing environment [77]. SEM has the advantage of being able test causal relationships among multiple variables in a model and to directly correct for measurement errors. We followed a two-step analytical approach as suggested by Anderson and Gerbing [78]. 

In the first step, we tested the adequacy of our measurement model by conducting a confirmatory factor analysis (CFA) including the latent variables agile work practices (T1), job demands (T2), job resources (T2), emotional fatigue (T2), and emotional engagement (T2). In the second step, we performed SEM to estimate the fit of the research model and to test our hypotheses. For both CFA and SEM, maximum-likelihood estimation with robust standard errors was employed, and the goodness-of-fit of the tested models was evaluated using the χ2 goodness-of-fit statistic, the Comparative Fit Index (CFI), the Tucker–Lewis-Index (TLI), the Root Mean Square Error of Approximation (RMSEA), and the Standardized Root Mean Square Residual (SRMR). Values larger than 0.95 for CFI and TLI, 0.08 or lower for RMSEA, and 0.10 or lower for SRMR indicate an acceptable model fit [79]. 

We preregistered our hypotheses and methods using the Open Science Framework OSF. The complete data set and evaluation code are also available in the website (https://osf.io/xpzbq, accessed on 22 December 2021).

## 5. Results

The descriptive statistics, including the means, standard deviations, inter-correlations of the study variables, and alpha reliabilities can be found in Table 1. 

### 5.1. Measurement Model

We first tested the measurement model consisting of five latent variables and their reflective indicators, which are (1) agile work practices with the four factors self-organized team work, iterative planning, incrementation, and retrospective; (2) job demands with workload, time pressure, and work interruptions; (3) job resources with autonomy, feedback from task, and peer support; (4) emotional fatigue, with three corresponding items; and (5) emotional engagement, with three corresponding items as indicators. The results of the CFA indicated that our hypothesized measurement model fitted the data well (χ2 (124) = 188.682, CFI = 0.97; TLI = 0.96, RMSEA = 0.05; SRMR = 0.05). Standardized coefficients from items to factors ranged from 0.45 to 0.92 and all indicators loaded significantly on their intended latent factors (*p* < 0.001), which supports the distinctiveness of the constructs [80].

### 5.2. Total Effects

We hypothesized that agile work practices are negatively related to emotional fatigue (Hypothesis 1) and that agile work practices are positively related to emotional engagement (Hypothesis 2). To estimate the total effects of agile work practices on occupational well-being, we tested our model, excluding job demands and job resources. The fit of the total effects model was satisfactory, as all criteria exceeded the established cut-offs (χ2 (32) = 46.965, CFI = 0.99; TLI = 0.98, RMSEA = 0.04; SRMR = 0.04). Agile work practices were significantly and negatively related to emotional fatigue (γ = −0.26, *p* = <0.001), whereas no significant relationship was found between agile work practices and emotional engagement (γ = 0.15, *p* = 0.095). The results provided support for Hypothesis 1, but not for Hypothesis 2.

### 5.3. Direct and Indirect Effects

**Figure 2:**
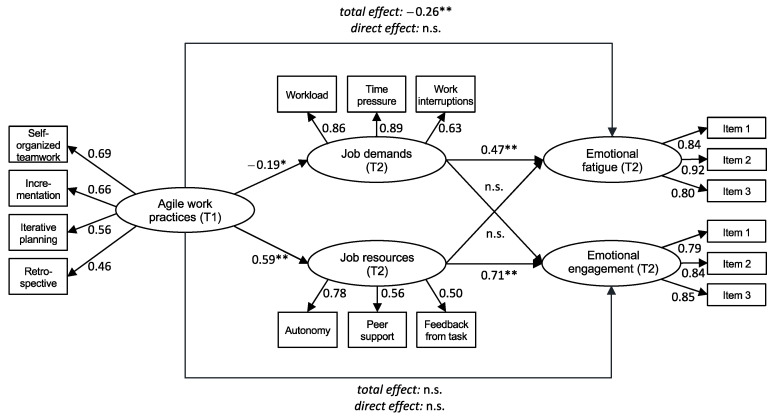
Structural equation model of the total, direct, and indirect effects of agile work practices, job demands, and job resources on emotional fatigue and emotional engagement (path coefficients are standardized. * *p* < 0.05, ** *p* < 0.01, n = 260).

We tested our model (Figure 1) including the two indirect paths from agile work practices to emotional fatigue via job demands, and from agile work practices to emotional engagement via job resources. The model showed an acceptable fit to the data in relation to our proposed reference values (χ2 (125) = 188.758, CFI = 0.97; TLI = 0.96, RMSEA = 0.05; SRMR = 0.05). Figure 2 presents the standardized parameter estimates for the hypothesized model. As expected, the path from agile work practices to job demands was negative and significant (γ = −0.19, *p* = 0.035), whereas the path from agile work practices to job resources was positive and significant (γ = 0.59, *p* < 0.001). Thus, our Hypotheses 3 and 4 were both supported by the results. 

Regarding indirect effects, we hypothesized that job demands mediate the negative relationship between agile work practices and emotional fatigue (Hypothesis 5) and that job resources mediate the positive relationship between agile work practices and emotional engagement (Hypothesis 6). The direct effect of job demands on emotional fatigue (γ = 0.47, *p* < 0.001) and of job resources on emotional engagement (γ = 0.71, *p* = 0.001) were positive and significant, whereas the complementary effect of job demands on emotional engagement and of job resources on emotional fatigue were not significant. Furthermore, the indirect effect of agile work practices on emotional fatigue via job demands was negative and significant (γ_indirect_ = −0.09, *p* = 0.042), and the indirect effect of agile work practices on emotional engagement via job resources was positive and significant (γ_indirect_ = 0.42, *p* = 0.004). Both direct paths from agile work practices to emotional fatigue and engagement were not significant. Bootstrapping results for all direct and indirect effects are presented in Table 2. Thus, Hypotheses 5 and 6 were also supported.

### 5.4. Supplemental Analyses

To test the robustness of our model, we expanded our model in three ways. The detailed results of the supplemental analyses can be found in Tables S2–S4 from https://osf.io/xpzbq (accessed on 22 December 2021). First, in addition to agile work practices, we added HPWS as a latent control variable with its nine reflective items in our model (see Table S2 from https://osf.io/xpzbq (accessed on 22 December 2021)). This supplemental model also had an acceptable fit to the data (χ2 (309) = 421.949, CFI = 0.96; TLI = 0.95, RMSEA = 0.04; SRMR = 0.06). However, compared to our main model, only the path from agile work practices to job resources was positive and significant (γ = 0.031, *p* = 0.029), whereas the path from agile work practices to job demands was not significant (γ = −0.11, *p* = 0.309). However, both indirect effects from agile work practices on emotional fatigue via job demands, and from agile work practices on emotional engagement via job resources, were not significant. Moreover, HPWS was positively related to job resources (γ = 0.48, *p* < 0.001) but not to job demands, and there were no significant indirect effects of HPWS on well-being via work characteristics. To conclude, only the direct effect of agile work practices on job resources was supported in this control model (Hypotheses 4), suggesting that this effect is the most robust effect that persists even when controlling for HPWS.

In a second supplemental model, we checked the robustness of effects by controlling for the T1 measures of our mediator and outcome variables (i.e., job demands, job resources, emotional fatigue, emotional engagement). This additional analysis explored possible lagged endogenous change effects of agile work practices on the mediator and outcome variables (see Table S3 from https://osf.io/xpzbq (accessed on 22 December 2021)). However, the model fit of this model was not sufficient as CFI and TLI did not meet the acceptance criteria (χ2 (441) = 1007.914, CFI = 0.88; TLI = 0.87, RMSEA = 0.07; SRMR = 0.07). Additionally, no significant associations were found between agile work practices and work characteristics or well-being. High intercorrelation between T1 and T2 measures of the mediator and outcome variables (see Table 1) suggest high stability of these variables. Thus, the present study contributes by explaining interindividual differences in these variables but not mean−level changes over time. 

In a third supplemental model, we controlled for language because significant differences between the English and German questionnaire were found for three study variables when comparing means. The model containing language as a control variable had an acceptable fit (χ2 (139) = 222.051, CFI = 0.96; TLI = 0.95, RMSEA = 0.05; SRMR = 0.06). The direct and indirect significant paths reported for the main model persisted when controlling for language (see Table S4 from https://osf.io/xpzbq (accessed on 22 December 2021)).

## 6. Discussion

In this study we investigated the relationship between agile work practices and occupational well-being based on the two-path approach suggested by JD–R theory [19]. Specifically, we tested whether links between agile work practices and well-being could be explained by indirect effects via job demands and resources. First, examination of the total effects revealed that our results only partially supported the hypotheses regarding the relationship between agile work practices and well-being, namely for the outcome emotional fatigue, but not for emotional engagement. Therefore, our main interest in this study comes even more to the fore. We wanted to explore the mediating role of work characteristics in the relationship between agile work practices and well-being. Our results support our hypotheses by showing that agile work practices are positively related to job resources (i.e., autonomy, peer support, and feedback from task) and negatively related to job demands (i.e., workload, time pressure, and work interruptions). Agile work practices thus seem to be significantly associated with work characteristics, in this case with perceptions of various positive and negative work characteristics. In addition, we found that agile work practices indirectly influence fatigue via job demands and that agile work practices indirectly influence engagement via job resources. The direct effects of agile work practices on well-being were not significant in our model. This suggests that the relationship between agile work practices and well-being is only indirect through job demands and resources and that the total effect for agile work practices on emotional fatigue can be explained by the indirect influence of job demands. 

In a supplementary analysis, we tested the extent to which the direct and indirect effects persisted when we included high-performance work systems in our model. In this case, only the positive relationship between agile work practices and job resources remained, but not the relationship with job demands, nor the indirect relationships with well-being. These results suggest that the positive association between agile work practices and resources is most robust in our model and persists even when other human resources practices are considered. Thus, the link to resources could potentially play a larger role in explaining occupational well-being in agile teams than the link to demands. This is consistent with our observation that most studies to date have focused on the positive and motivational outcomes of agile work practices.

Although this was not the primary focus of our study, we were able to confirm the two main underlying processes suggested in JD−R theory: job demands are positively related to emotional fatigue, and job resources are positively related to emotional engagement [37]. Based on results from previous research [38,81], we also included and tested the paths from job demands to emotional engagement as well as from job resources to emotional fatigue in our model. Our results, however, did not confirm these two cross-paths. Looking at the further development of JD−R theory, namely the proposal to distinguish job demands into challenge and hindrance demands [82,83], may help to understand the nonsignificant relationship between job demands and engagement. Hindrance demands are those that are perceived as threatening and energy draining and are negatively related to employee engagement, whereas challenge demands are stressful demands that are simultaneously energy draining and stimulating and therefore positively related to engagement. Our construct of job demands contains both hindering (i.e., work interruptions) and challenging aspects of demands (i.e., workload and time pressure), which may explain the non-significant finding. The nonsignificant relationship between work resources and emotional fatigue may also be explained by previous research. Frone and colleagues [21] showed that the job resource autonomy was only related to the cognitive and physical aspects of fatigue but not to the emotional facet. In addition, resources such as feedback, social support, and job control were mainly found to be related to the burnout facets of cynicism and professional efficacy, but not as strongly to emotional exhaustion [84].

### 6.1. Theoretical and Practical Implications

Our findings make several important contributions to the literature. First, our primary motivation for this study was to investigate whether and how agile work practices are associated with occupational well-being. Initial research in the field of agile work practices suggests that agile work practices can lead to positive outcomes in terms of well-being due to their engaging potentials [28,85]. Although the potential of agile work practices to reduce stress and fatigue has been discussed [14,15], this perspective has not yet been systematically studied. Compared to previous studies, we included two different well-being outcomes in our model, emotional fatigue and emotional engagement, and we simultaneously examined the two different pathways leading to occupational well-being as suggested by JD−R theory. In terms of total effects, our results supported preliminary findings of a stress-reducing effect of agile teamwork [14,15], leading to lower levels of fatigue. However, the fact that we did not find a total effect on emotional engagement suggests that engagement, which is a positive and fulfilling state at work [37], does not simply result from the adoption of agile work practices. For this reason, the question of the role of work characteristics, which was the focus of our study, is an essential one. To date, fewer than a handful of studies have empirically studied the field of agile work practices, work characteristics, and positive outcomes at work, such as commitment or job satisfaction [12,14,15]. Moreover, none of the mentioned authors investigated the possible indirect effects between agile work practices and well-being via work characteristics but only focused on describing direct effects on work characteristics or well-being. 

Therefore, second, we explored these indirect effects, more specifically, to show that agile work practices indirectly affect well-being through the two different hypothesized pathways. On the one hand, agile work practices can reduce the risk of emotional fatigue by decreasing potential job demands. Specifically, our results suggest that self-organization, joint team planning, and prioritization of tasks at the beginning of agile iterations can better contain demands, such as workload, time pressure, and work interruptions [14,16]. Moreover, the incremental and iterative approach provides teams with a way to focus and work toward intermediate goals without getting lost in too much time pressure or task overload. This potential of agile working methods has hardly been discussed so far, and if so, then only qualitatively [16]. However, our results show that it should not be underestimated. Agile work practices increase emotional engagement by fostering important job resources. For example, individuals in agile teams experience a high level of autonomy through self-organization and decision-making power in contrast to classic hierarchical environments [30,60]. Agile work practices also promote social cohesion and mutual support as team members take shared responsibility and regularly reflect on the process and collaboration within the team [56]. Furthermore, feedback plays a central role for agile teams as agile work practices provide many opportunities for feedback [12,62,86]. We found empirical support for the association between agile work practices and important job resources, which suggests that these practices may meet three innate needs of human beings, which are autonomy, belonging, and competence [11]. This supports the assumption that agile work practices provide a framework for more humane, motivating, and fulfilling work [28].

Third, we examined the relationships between agile work practices and occupational well-being in a systematic and rigorous methodological way based on sound psychological theory. Thus, we were able to close the previously large gap between practical literature from software development and psychological theory. Furthermore, we addressed methodological limitations of previous research by basing our results on a very heterogeneous sample, with participants from different countries, cultures, and industries, and by following a two-measurement-point approach.

Fourth, we also add to the literature of JD−R theory by examining concrete practices related to specific job demands and resources. To date, research on this model has often focused on discussing only individual perceptions of work characteristics as predictors of well-being but less on incorporating specific work practices as effects at organizational or team level [22]. This should also be an impetus for more intensive research into the predictors of work characteristics. Just as we examined agile work practices as a predictor of work design and consequently well-being, there could be further streams of research to link the theoretical framework of the JD−R model even more closely with practical approaches (see also [87], for example).

Fifth, from the perspective of practitioners, this paper provides important implications for management, organizational development, and human resources. Agile work practices are becoming more widespread in organizations [27]. On the one hand, agile work practices and their autonomous and empowering way of working seem to be very attractive for employees [13]. On the other hand, agile work practices were shown to be positively related to employees’ mental health. It is of significant contribution to better understand how and why agile work practices affect employee well-being and how to design agile work practices and agile environments so that they can reach their full potential with respect to employee well-being. Our findings suggest that both job demands and job resources should be considered when adopting agile work practices. For example, attention should be paid to ensuring that resources such as autonomy, social support, and feedback are strengthened in the new setting. Furthermore, it is important to give agile teams the autonomy and control to manage certain demands themselves, such as workload and time pressure, and to ensure that the teams can work without much work interruptions during the iterations to prevent overload and exhaustion. From a business point of view, increasing the well-being is not only an appreciative intention, but also the intention to improve organizational performance and financial results. High job demands are associated with negative consequences such as absenteeism, illness, work−home interference, or turnover intentions, whereas job resources are related to organizational commitment, job satisfaction, innovation, and performance [38]. 

### 6.2. Limitations and Future Research

A few limitations of the present study should be noted. First, although we collected our measures at two different measurement times, which may have reduced common-method bias, there could still be a potential bias in the data given the use of self-reported questionnaires [65]. Future research could complement self-reports with other measurement methods, such as ratings provided from different perspectives (e.g., company leaders or customers) or objective well-being measures (e.g., days of sick leave). With a multi-method approach, error influences of the single measurement method are compensated by measuring the same construct with a different method. In addition to quantitative data, qualitative data should also be collected to support and to further differentiate our results. In particular, the results of a content analysis based on interview data with open questions might help to better understand and interpret the underlying processes and provide clues for the direction of future research, e.g., which individual or conceptual factors can strengthen or mitigate the relationships included in our model.

Second, we were also unable to detect changes in our mediator and outcome variables, as these variables were very stable across the two measurement time points. Thus, the results of the study do not allow us to draw conclusions about causality. Future research should adopt a longitudinal design with three or ideally more measurement waves [88] to provide the opportunity to gain greater insight into the reported effects and their potential causal influence. In the context of our research question, it would be interesting to follow teams as they transition to agile ways of working over, for example, a one-year period and measure the changes in perceptions of work characteristics and well-being at different measurement points during that year. In such an intervention study, a control group that does not undergo any changes should be introduced to further strengthen the robustness of our results. It would also be a good approach to include entire teams in the sample and to investigate the changes at team level in comparison to individual changes via a multilevel approach. 

Third, we surveyed a very broad population of employees (different countries, organizations, and industries) and showed that the results persist even when controlling for language differences in the questionnaire (German vs. English). Heterogeneity could thus be guaranteed for some aspects, but not for all. For example, all our participants were software development professionals. However, agile work practices not only are nowadays a common practice in the software industry but have been applied in various business areas [2]. Following the suggestion of Junker et al. [5], it would be interesting to see in which contexts agile work practices have positive outcomes for individual well-being and in which contexts they might not. Study results have already shown that the agile way of working can be beneficial for teams beyond IT by being positively associated with the psychological safety climate, engagement, and performance of various functional areas of a bank [43]. However, it also has been discussed that agile teamwork does not necessarily unfold the benefits in every context [89]. Thus, replications of our study for different industries, groups of professionals, and cultural backgrounds of companies may help to make more nuanced, contextual statements here.

Fourth, in the theoretical part of this paper, we have already discussed a lack of consensus in the research field on how agile work practices should be defined and measured. We might not have captured all relevant dimensions of the concept, as agile work practices is a broader collection of practices than the four practices we have included. For example, agile work practices, which have been included in other studies but not in ours due to inconsistencies in measurement concept, include daily stand-up meetings [12], customer relation and collaboration [13,14], use of burndown charts [12], or continuous orientation [14]. The ambiguity about the construct of agile work practices calls for a systematic review of existing concepts and for a psychometric validation study to develop a valid measurement concept that can be used consistently across different studies. Only in this way can it be guaranteed that the results of different studies can be compared and integrated. With the systematic elaboration of a behavioral taxonomy of agility, researchers have already provided a starting point to develop a unified instrument with regards to workforce agility [3,90]. Moreover, it is not necessarily the case that the same results can be replicated for the agile work practices we have not included. It may even be that the opposite is the case for some practices. Thus, it should be further explored in future research to determine the extent to which agile work practices can also reverse and pose risks to well-being [91]. For example, the frequent cooperation with customers is an essential feature for the success of agile development projects, which could cause additional stress, for example, when promises are made that cannot be kept [92]. Feelings of control through a high level of transparency and progress tracking as well as the risk of self-exploitation can be stress hazards in agile teams [16]. Pressure to perform, not to disappoint but to prove themselves to the team, could also be negatively related with well-being [62]. 

Fifth, the timing of data collection should also be considered somewhat critically. The data were collected during the first six months of the COVID-19 pandemic. The risks and uncertainties posed by the pandemic also have a direct impact on employee well-being [93,94]. After all, 20.8% of the study participants stated that their ability to work is strongly affected and 13.8% agreed that they are worried about their job because of the Corona crisis. However, research has shown that agile project management acted as a buffering job resource in the relationship between COVID-19 demands and emotional exhaustion [95]. From this, it can be assumed that the pandemic situation has strengthened the effects rather than weakened them. Nevertheless, it would certainly be advisable to repeat the study at a time that is not affected by a pandemic.

Sixth, we focused on investigating the direct and indirect effects of agile work practices, work characteristics, and well-being. What we did not show in our study, but what has been relatively stable results in the past, is the interaction effects of job demands and job resources on well-being proposed by the JD−R model [38]. Future studies should also consider this interaction effect, i.e., to what extent the interaction of job demands and job resources affects well-being in the context of agile work practices. It can be assumed that emotional fatigue will be highest for a high level of job demands and a low level of job resources, and that a high level of job resources can buffer the stress effect of job demands on negative outcomes. With a cross-over effect on engagement, it would be reasonable to assume that emotional engagement is lowest when job demands are high and job resources are low. However, there is no clear evidence in the other direction, as it is assumed that an intermediate level of job demands might be the optimum, not a low one.

Finally, what also remains open is the extent to which other variables, in particular personnel resources or the organizational context, can interact with job demands and job resources and thus influences the relationship between agile work practices and occupational well-being. For successful agile work, organizations need to create an environment with an autonomous, open, and collective culture [90,96]. Instead of a directive, command-and-control style of leadership and micro-management, managers need to have trust that the team will make the right decisions [62,97]. Agile leaders need to be able to create a climate to support innovation and psychological safety, so individuals can share information, support each other, and come up with creative ideas [24]. In addition, the organizational structure must be adapted to the structure of self-managing teams, which are more likely to be effective in low-hierarchical, decentralized organizations with less explicit rules, policies, and procedures [29,30]. Regarding individual factors, previous studies showed, for example, how agility is related to extraversion [98,99], interpersonal skills, and sociability [100]. By addressing in future research how personal and organizational resources potentially influence the stress and motivation process in agile teams, it could be better understood why some employees in agile teams are apparently more resistant to strain and more engaged than others who work in the same environment and role.

## 7. Conclusions

Subjective well-being of employees is becoming increasingly important in organizations, and it is nowadays recognized that well-being is a critical factor in human functioning and job performance [101]. Based on JD−R theory [19,37], we developed and tested a theoretical model to examine the relationships between agile work practices, work characteristics, and occupational well-being. In summary, we found that agile work practices are positively and indirectly related to occupational well-being via work characteristics, in particular job demands and job resources. On the one hand, agile work practices aim at a sustainable pace [14] as well as a clear work focus [23]. Thus, energy-depleting job demands that are positively related with emotional fatigue, such as workload, time pressure, or work interruptions, can be reduced by agile work practices. On the other hand, agile work practices are designed to foster work characteristics identified as important job resources, which are positively associated with emotional engagement, such as autonomy, feedback, and social support [54,55,56]. On a practical level, our findings indicate that for successful implementation of agile work practices, organizations need to consider the impact of job demands and job resources to strengthen employee well-being in the agile work context. Resources related to agile work practices need to be fostered, and certain stressful demands can be reduced.

## Figures and Tables

**Table 1:** Item means, item standard deviations, and correlations among the study variables. n = 260 employees.

	*M*	SD	1	2	3	4	5	6	7	8	9	10	11	12
**Agile work practices**														
1 Self-organized teamwork (T1)	4.16	0.72	(0.80)											
2 Incrementation (T1)	3.89	0.77	0.45 **	(0.68)										
3 Iterative planning (T1)	4.22	0.81	0.39 **	0.35 **	(0.78)									
4 Retrospectives (T1)	4.32	0.96	0.29 **	0.29 **	0.38 **	(0.94)								
5 High-performance work systems (T1)	3.54	0.65	0.37 **	0.34 **	0.24 **	0.14 *	(0.80)							
**Job demands**														
6 Workload (T1)	3.42	0.89	−0.14 *	−0.13	−0.10	−0.10	−0.11	(0.82)						
7 Time pressure (T1)	2.83	1.05	−0.20 **	−0.18 **	−0.22 **	−0.12	−0.16 *	0.75 **	(0.91)					
8 Work interruptions (T1)	3.37	0.95	−0.15 *	−0.22 **	−0.10	−0.09	−0.12 *	0.52 **	0.53 **	(0.80)				
9 Workload (T2)	3.39	0.90	−0.07	−0.10	−0.06	−0.11	−0.06	0.76 **	0.60 **	0.46 **	(0.82)			
10 Time pressure (T2)	2.87	1.05	−0.12	−0.12	−0.08	−0.16 *	−0.18 **	0.65 **	0.67 **	0.46 **	0.77 **	(0.90)		
11 Work interruptions (T2)	3.30	0.98	−0.01	−0.12	−0.04	0.02	−0.07	0.48 **	0.50 **	0.65 **	0.54 **	0.56 **	(0.81)	
**Job resources**														
12 Decision-making autonomy (T1)	4.02	0.81	0.41 **	0.29 **	0.24 **	0.08	0.39 **	0.03	−0.12	0.01	0.06	−0.10	0.07	(0.89)
13 Work scheduling autonomy (T1)	3.97	0.80	0.36 **	0.25 **	0.27 **	0.16 **	0.28 **	−0.19 **	−0.26 **	−0.15 *	−0.15 *	−0.28 **	−0.09	0.56 **
14 Work method autonomy (T1)	4.08	0.83	0.37 **	0.35 **	0.25 **	0.15 *	0.33 **	−0.06	−0.14 *	−0.06	−0.04	−0.15 *	0.02	0.75 **
15 Peer support (T1)	4.13	0.82	0.37 **	0.27 **	0.25 **	0.19 **	0.48 **	−0.10	−0.17 **	−0.16 **	−0.06	−0.12	−0.13 *	0.38 **
16 Feedback from task (T1)	3.24	0.84	0.13*	0.32 **	0.17 **	0.04	0.42 **	0.03	−0.11	−0.15 *	−0.05	−0.11	−0.16 *	0.26 **
17 Decision-making autonomy (T2)	4.04	0.88	0.32 **	0.31 **	0.20 **	0.11	0.37 **	0.04	−0.09	−0.02	0.05	−0.12	0.05	0.68 **
18 Work scheduling autonomy (T2)	4.01	0.72	0.27 **	0.29 **	0.21 **	0.11	0.29 **	−0.09	−0.19 **	−0.11	−0.09	−0.21 **	−0.07	0.51 **
19 Work method autonomy (T2)	4.09	0.87	0.33 **	0.36 **	0.19 **	0.14 *	0.31 **	−0.04	−0.14 *	−0.08	−0.07	−0.18 **	−0.05	0.59 **
20 Peer support (T2)	4.09	0.81	0.31 **	0.21 ***	0.17 **	0.15 *	0.39 **	−0.11	−0.14 *	−0.11	−0.10	−0.19 **	−0.11	0.37 **
21 Feedback from task (T2)	3.25	0.93	0.09	0.21 **	0.08	−0.01	0.31 **	0.03	−0.05	−0.11	0.06	−0.04	−0.13 *	0.24 **
**Occupational well-being**														
22 Emotional fatigue (T1)	2.54	1.06	−0.14 *	−0.16 **	−0.11	−0.03	−0.07	0.31 **	0.32 **	0.26 **	0.27 **	0.26 **	0.26 **	−0.09
23 Emotional engagement (T1)	4.05	0.81	0.22 **	0.22 **	0.21 **	0.05	0.38 **	0.04	−0.05	−0.16 **	0.03	−0.10	−0.14 *	0.40 **
24 Emotional fatigue (T2)	2.80	1.10	−0.19 **	−0.17 **	−0.05	−0.14 *	−0.20 **	0.40 **	0.34 **	0.28 **	0.38 **	0.42 **	0.38 **	−0.10
25 Emotional engagement (T2)	3.85	0.85	0.07	0.15*	0.16*	−0.03	0.27 **	0.00	−0.07	−0.15*	0.05	−0.08	−0.18 **	0.36 **
	**13**	**14**	**15**	**16**	**17**	**18**	**19**	**20**	**21**	**22**	**23**	**24**	**25**	
**Agile work practices**														
1 Self−organized teamwork (T1)														
2 Incrementation (T1)														
3 Iterative planning (T1)														
4 Retrospectives (T1)														
**5 High-performance work systems (T1)**														
**Job demands**														
6 Workload (T1)														
7 Time pressure (T1)														
8 Work interruptions (T1)														
9 Workload (T2)														
10 Time pressure (T2)														
11 Work interruptions (T2)														
**Job resources**														
12 Decision−making autonomy (T1)														
13 Work scheduling autonomy (T1)	(0.84)													
14 Work method autonomy (T1)	0.57 **	(0.91)												
15 Peer support (T1)	0.25 **	0.34 **	(0.89)											
16 Feedback from task (T1)	0.25 **	0.27 **	0.45 **	(0.81)										
17 Decision−making autonomy (T2)	0.50 **	0.68 **	0.26 **	0.27 **	(0.92)									
18 Work scheduling autonomy (T2)	0.61 **	0.54 **	0.22 **	0.28 **	0.67 **	(0.84)								
19 Work method autonomy (T2)	0.53 **	0.71 **	0.24 **	0.25 **	0.81 **	0.67 **	(0.93)							
20 Peer support (T2)	0.30 **	0.40 **	0.68 **	0.33 **	0.42 **	0.30 **	0.39 **	(0.90)						
21 Feedback from task (T2)	0.14 *	0.21 **	0.27 **	0.51 **	0.36 **	0.33 **	0.32 **	0.27 **	(0.89)					
**Occupational well-being**														
22 Emotional fatigue (T1)	−0.15 **	−0.11	−0.26 **	−0.17 **	−0.11	−0.10	−0.21 **	−0.22 **	−0.02	(0.88)				
23 Emotional engagement (T1)	0.30 **	0.36 **	0.41 **	0.43 **	0.36 **	0.28 **	0.34 **	0.32 **	0.38 **	−0.23 **	(0.87)			
24 Emotional fatigue (T2)	−0.16 **	−0.10	−0.27 **	−0.18 **	−0.11	−0.17 **	−0.18 **	−0.25 **	−0.12	0.66 **	−0.22 **	(0.89)		
25 Emotional engagement (T2)	0.24 **	0.31 **	0.31 **	0.35 **	0.37 **	0.31 **	0.33 **	0.25 **	0.37 **	−0.18 **	0.64 **	−0.22 **	(0.87)	

Note. Cronbach’s α on the diagonal. * *p* < 0.05, ** *p* < 0.01.

**Table 2:** Direct and indirect effects using bootstrapping (10,000 replications).

	Bootstrap			
Direct Effects	*Est.*	*SE*	*p*	*CI 95%*
Agile work practices T1 → Emotional fatigue T2	−0.05	0.21	0.623	(−0.55, 0.29)
Agile work practices T1 → Emotional engagement T2	−0.24	0.21	0.095	(−0.71, 0.13)
Agile work practices T1 → Job demands T2	−0.19	0.11	0.035	(−0.42, 0.02)
Agile work practices T1 → Job resources T2	0.59	0.16	< 0.001	(0.43, 10.06)
Job demands T2 → Emotional fatigue T2	0.47	0.12	< 0.001	(0.49, 0.94)
Job demands T2 → Emotional engagement T2	0.01	0.10	0.918	(−0.18, 0.19)
Job resources T2 → Emotional fatigue T2	−0.19	0.20	0.155	(−0.65, 0.16)
Job resources T2 → Emotional engagement T2	0.71	0.25	0.001	(0.26, 10.24)
**Indirect Effects**	***Est.***	***SE***	***p***	***CI 95%***
Agile work practices T1 → Job demands T2 → Emotional fatigue T2	−0.09	0.09	0.042	(−0.31, 0.03)
Agile work practices T1 → Job resources T2 → Emotional fatigue T2	−0.11	0.16	0.166	(−0.49, 0.13)
Agile work practices T1 → Job demands T2 → Emotional engagement T2	0.00	0.03	0.928	(−0.05, 0.05)
Agile work practices T1 → Job resources T2 → Emotional engagement T2	0.42	0.22	0.004	(0.10, 0.95)

Note. All parameter estimates are presented as standardized coefficients. *Est.* = estimation, *SE* = standard error, *p* = significance level, *CI* = confidence interval, H = hypothesis, T = measuring time. n = 260.

## Data Availability

Data supporting these findings is available via https://osf.io/xpzbq accessed on 22 December 2021.
